# Efficacy of adipose derived stem cells on functional and neurological improvement following ischemic stroke: a systematic review and meta-analysis

**DOI:** 10.1186/s12883-020-01865-3

**Published:** 2020-08-10

**Authors:** Mahmoud Yousefifard, Jebreil Shamseddin, Asrin Babahajian, Arash Sarveazad

**Affiliations:** 1 Physiology Research Center, Iran University of Medical Sciences, Tehran, Iran; 2 Infectious and Tropical Diseases Research Center, Hormozgan Health Institute, Hormozgan University of Medical Sciences, Bandar Abbas, Iran; 3 Liver and Digestive Research Center, Research Institute for Health Development, Kurdistan University of Medical Sciences, Sanandaj, Iran; 4 Colorectal Research Center, Iran University of Medical Sciences, Tehran, Iran; 5 Nursing Care Research Center, Iran University of Medical Sciences, Tehran, Iran

**Keywords:** Stem cells, Stroke, Functional recovery

## Abstract

**Background:**

The evidence on the efficacy of adipose derived stem cells (ADSCs) in the treatment of stroke is controversial. Therefore, the aim of present systematic review and meta-analysis is to evaluate the efficacy of ADSCs administration in the treatment of animal models of ischemic stroke.

**Methods:**

An extensive search was performed on electronic databases of Medline, Embase, Scopus, CENTRAL and Web of Science until December 31, 2018. Animal studies that used ADSCs in treatment of ischemic stroke were included. The data were recorded as mean and standard deviation and then a pooled standardized mean difference (SMD) with 95% confidence interval (95% CI) was reported.

**Results:**

Twenty articles were included in the present meta-analysis. It was observed that administration of ADSCs improves motor function (SMD = 2.52, 95% CI: 1.67 to 3.37, *p* < 0.0001) and neurological status (SMD = 2.05, 95% CI: 1.33 to 2.78, *p* < 0.0001) in animals following an ischemic stroke. Multivariate meta-regression showed the model of stroke induction (*p* = 0.017) and the number of transplanted cells (*p* = 0.007) affect the efficacy of ADSCs administration on motor function improvement following the stroke.

**Conclusion:**

Moderate to high levels of evidence indicate a strong efficacy of ADSCs transplantation on motor function and neurological improvement following ischemic stroke in animal models. However, no reports regarding the dose-response effect of ADSCs administration on stroke exist in the literature. As a result, further pre-clinical studies are recommended to be conducted on the matter.

## Background

Stroke is a medical emergency that is defined as a set of short and long-term clinical signs and symptoms indicative of a partial or whole brain damage [1]. Stroke is the second cause of death (250 to 400 deaths per 100,000 every year) and one of the main causes of long-term disability in the world [2–4]. However, there are no protective or restorative treatments for stroke, particularly in the retrieval of the lost nervous tissue. Common treatments for stroke include intravenous thrombolytics, tissue plasminogen activator (tPA) [5] and endovascular treatment [6].

The use of stem cells in regenerative medicine has been suggested in several pre-clinical [7–10] and clinical studies [11–13] for nervous system disorders such as spinal cord injury, Alzheimer’s disease, Parkinson’s and stroke. In this regard, mesenchymal stem cells (MSCs) have become the focus of attention of researchers, because of their ease of access, pluripotency, ability to secrete various growth factors and ethical approval in clinical applications [14]. One of the sources of MSCs is the adipose tissue [15, 16]. Adipose derived stem cells (ADSCs) may be beneficial in the repair of neurological lesions such as stroke, because these cells are abundantly available, can be accessed and collected using a minimally-invasive technique [15, 17] and secret many growth factors such as NGF, BDNF, GDNF [18, 19] and VEGF [20]. Therefore, ADSCs administration have been proposed recently as a new therapeutic option in stroke management and its treatment [21, 22]. Meanwhile, articles studying the effects of ADSCs administration on the outcomes of stroke, including motor function and neurologic status, report controversial results, so that a definitive statement on this matter is yet to be achieved. On one hand, pre-clinical studies have shown the efficacy of stem cell transplantation in the treatment of stroke. However, recent clinical trials have shown that although stem cells therapy is safe, its efficacy in the management of stroke is still questionable [23–25]. This failure in confirming the therapy as an efficient treatment option in managing stroke may be due to the major differences in the treatment protocols proposed by preclinical studies. In other words, there is no consensus over the optimum dose of stem cell therapy, its best route of administration, most efficient therapeutic time window, best source of stem cells and etc. Therefore, the aim of the present systematic review and meta-analysis is to thoroughly evaluate the existing evidence regarding the efficacy of ADSCs administration in the improvement of motor function and neurologic status in animal models of ischemic stroke, compared to no-treated animals.

## Methods

### Study design

In this study, the efficacy of ADSCs in the treatment of ischemic stroke was evaluated by collecting published data in electronic database and gray literature. We used a similar method to our previous studies to perform the databases searches and summarizing the data [26–35]. A brief description of the search and data extraction methods is presented as follows.

### PICO definition

PICO in the present study was defined as: Problem (P): animal models for ischemic stroke, intervention (I): administration of ADSCs, comparison (C): with ischemic stroke model without any treatment; outcome (O): motor function and neurologic assessment.

### Search strategy

The search strategy consisted of three steps: a) systematic search in databases b) manual search on Google and Google Scholar search engines and c) review of the bibliography of the related articles.

Initially, under the supervision of an expert researcher in the field of stroke and stem cells, using the MeSh and Emtree terms of Medline and Embase databases, respectively, and by screening titles and abstracts of the related articles, relevant keywords were extracted. Then, with the advice of an expert librarian, familiar with the search, and using the standard tags for each database, the search strategy was defined separately for, Medline, Embase, Scopus, Web of Science and CENTRAL databases and the search was performed until December 31st, 2018. The search term in the Medline database is presented in Table 1.

**Table 1:** Search strategy for screening of Medline records via PubMed

((“Stroke”[mh] OR “Brain Infarction”[mh] OR “Stroke Rehabilitation”[mh] OR “Brain Stem Infarctions”[mh] OR “Infarction, Anterior Cerebral Artery”[mh] OR “Cerebral Infarction”[mh] OR “Reperfusion Injury”[mh] OR “Hypoxia-Ischemia, Brain”[mh] OR “Brain Ischemia”[mh] OR “Stroke”[tiab] OR “Brain Infarction”[tiab] OR “Stroke Rehabilitation”[tiab] OR “Brain Stem Infarctions”[tiab] OR “Infarction, Anterior Cerebral Artery”[tiab] OR “Cerebral Infarction”[tiab] OR “Reperfusion Injury”[tiab] OR “Hypoxia-Ischemia, Brain”[tiab] OR “Brain Ischemia”[tiab] OR “Cerebrovascular Accident”[tiab] OR “Brain Vascular Accident”[tiab] OR “Cerebrovascular Stroke”[tiab])) AND (“Adipose-derived Stem Cells”[mh] OR “Adipose-derived Stem Cells”[tiab] OR “Human Adipose-derived Stem Cells”[tiab] OR “The potential of adipose stem cells”[tiab] OR “Adult Stem Cells derived from adipose tissue”[tiab] OR “Stem cells from fat”[tiab] OR “Stem cells from adipose tissue”[tiab] OR “Adipose Stem Cell”[tiab] OR “Fat tissue stem cells”[tiab] OR “Stem cells from adipose tissue”[tiab] OR “Adipose Tissue Derived Multipotent Mesenchymal Stromal Cells”[tiab] OR “Brown Adipose Tissue Derived Stem Cells”[tiab] OR “Autologous Adipose Tissue Derived Mesenchymal Stem Cells”[tiab] OR “Adipose tissue stem cells”[tiab] OR “Adipose Tissue-Derived Stem Cells”[tiab] OR “Stromal Stem Cells from Human Adipose Tissue”[tiab] OR “Adipose-Derived Mesenchymal Stem Cells”[tiab] OR “Human mesenchymal stem cells derived from adipose tissue”[tiab] OR “Adipose tissue stem cells”[tiab] OR “Cells from fat”[tiab] OR “Adipose derived Mesenchymal stem cells”[tiab] OR “Mesenchymal Stem Cells from Adipose Tissue”[tiab] OR “Adipose tissue stem cells”[tiab] OR “Adipose Tissue-Derived Mesenchymal Stem Cells”[tiab] OR “Adipose Tissue Stem Cells”[tiab] OR “Stromal cells from the adipose tissue”[tiab] OR “Stem cells derived from various mesenchymal tissues:”[tiab] OR “Adipose-derived adult stem cells”[tiab] OR “Stem cells from mouse adipose tissue”[tiab] OR “Adipose tissue mesenchymal stem cells”[tiab] OR “Adipogenic”[tiab] OR “Adipogenesis”[tiab] OR “Adiposytes”[tiab])	

### Selection criteria

All experimental (animals) studies that used ADSCs in the treatment of ischemic stroke were included. Studies without a control group (non-treatment group), studies investigating about hemorrhagic stroke, studies lacking the assessment of functional or neurological status, and review studies were excluded.

Ischemic stroke models in eligible studies were achieved using middle cerebral artery occlusion (MCAO) and common carotid artery occlusion (carotid clamp model) methods. MCAO is an ischemic model to simulate cerebrovascular embolic events, and carotid clamp simulates a systemic hypo-perfusion situation such as in myocardial infarction or hemorrhagic shock. The details of inducing the models were presented in previous studies [36, 37].

### Data collection and risk of bias assessment

After searching the databases, achieved records were entered to EndNote software (version X7, Thomson Reuters, 2014) and duplicate articles were eliminated. In the first stage, two independent reviewers screened titles and abstracts. After identifying the potentially relevant articles, the full texts of the articles were received and assessed in detail. After selecting the inclided articles, relevant data of the articles were entered into a pre-designed checklist based on the PRISMA statement [38]. Any disagreement was resolved by discussion with a third reviewer. The article characteristics (name of the first author and year of publication), animal species, weight, stroke induction model, route of ADSCs transplantation, number of animals, motor function or neurological assessments in follow up evaluations, interval times of follow up and possible sources of bias in the articles were recorded in the checklist. The outcomes included motor function assessment and neurological status. If the results were presented in graphs, the values were extracted using Plot Digitizer software (Version 2.0). The quality control of the articles was performed based on the STAIR Criteria [39].

### Statistics

Statistical analysis was performed using STATA software version 14.0 (Stata Corporation, College Station, TX). All data were recorded as mean and standard deviation and then, using “metan” command, a pooled standardized mean difference (SMD) with a 95% confidence interval (95% CI) was calculated. Heterogeneity between the studies was evaluated using I^2^ tests and *p* value less than 0.1 was considered significant (representing heterogeneity). In case of homogeneity, fixed effect model was used, and in case of heterogeneity, a subgroup analysis was performed to determine the source of heterogeneity. A multivariate meta-regression analysis was performed to assess any possible collinearity in the significant variable in subgroup analyses. If the source of heterogeneity was unknown, random effect model was used. Before performing the overall meta-analysis, we performed a meta-regression analysis to assess the effect of follow up duration on efficacy of ADSCs in motor and neurologic improvement. Since the follow-up duration did not affect the efficacy of the ADSCs therapy on motor function (coefficient = − 0.006; *p* = 0.900) and neurological improvement (coefficient = − 0.017; *p* = 0.504) (Figure S1), we pooled the data. Begg’s test was used to identify the publication bias [40].

## Results

### Characteristics

**Fig. 1:**
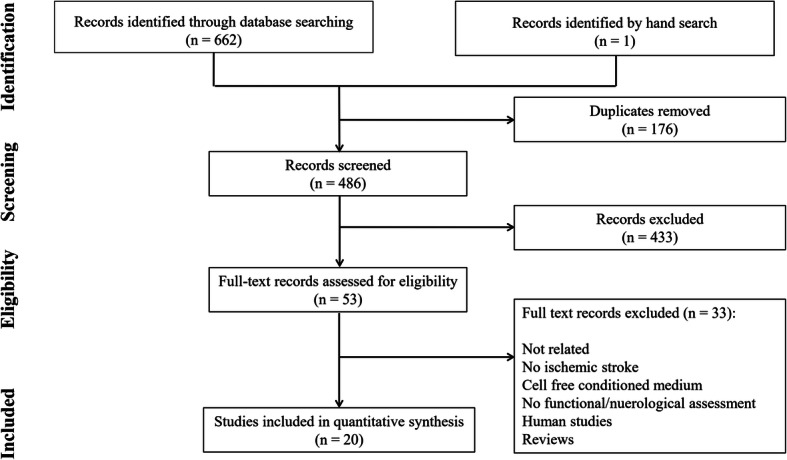
PRISMA flow diagram of the present meta-analysis

Four hundred eighty-six non-duplicated records were found. After screening and assessment of potentially relevant studies, 20 articles were entered into the meta-analysis [41–60] (Fig. 1). Sixteen studies were performed on rats and 4 on mice. Sixteen studies used the MCAO model to induce ischemic stroke And the other four studies used carotid clamp. The duration of occlusion was 90 min in 7 studies, 60 min in 2 studies, less than 60 min in 4 studies, 180 min in 1 study, 120 min in 1 study and permanent in 5 studies. In 7 studies, ADSCs were transplanted immediately after arterial occlusion, in 5 studies were injected between 30 min to 3 h after the occlusion and in 5 studies were administered 24 h after the stroke. Also, in three studies, ADSCs was transplanted between 168 to 1008 h after stroke. The route of cell administration was intravenous in 12 studies, intracranial in 6 studies and intra-arterial in 2 studies. The type of graft was xenograft in 10 studies, allograft in 7 studies and autograft in 3 studies. The number of transplanted ADSCs in most studies (15 studies) ranged from 5 × 10^5^ to 2 × 10^6^. Furthermore, the length of animal follow-up varied between 1 and 70 days, being 28 days in 7 studies. Table 2 summarizes the characteristics of the included articles.

**Table 2:** Summary of included studies

Author; Year	Sample size (control/treated)	Gender; Strain; Species; weight	Duration of occlusion (minute)	Model	Stroke to treatment interval (hours)	Type of administration	Type of graft	Number of transplanted cells	Outcome	Follow up duration (day)
Chen; 2016 [41]	12 / 12	Male; Sprague-Dawley; Rat; 350–375 g	50	MCAO	3	IV	Xenograft	1.2 × 10^6^	Neuorologic	28
Chi; 2016 [42]	3 / 3	NR; BALB/c; Mice; 25 g	Permanent	MCAO with thrombin	2	IC	Xenograft	1 × 10^6^	Motor	14
Chung; 2015 [43]	5 / 5	Male; Sprague-Dawley; Rat; 250–300 g	7	Carotid clamp	0	IV	Xenograft	1 × 10^6^	Motor	7
Chung; 2017 [44]	8 / 8	Male; Sprague-Dawley; Rat; 250–300 g	7	Carotid clamp	0	IV	Xenograft	1 × 10^6^	Motor	7
Du; 2014 [45]	20 / 60	Male; Sprague-Dawley; Rat; 260–280 g	90	Carotid clamp	24	IC/IA/IV	Allograft	5 × 10^5^	Neuorologic	21
Ghazavi; 2017 [46]	16 / 8	Male; Wistar; Rat; 220–270 g	30	MCAO	0.5	IV	Allograft	2 × 10^6^	Motor	1
Gutiérrez-Fernández; 2013 [47]	10 / 10	Male; Sprague-Dawley; Rat; 250–300 g	Permanent	MCAO	0.5	IV	Allograft	2 × 10^6^	Motor	14
Gutiérrez-Fernández; 2015 [48]	15 / 30	Male; Sprague-Dawley; Rat; 250–320 g	Permanent	MCAO	0.5	IV	Xenograft/Allograft	2 × 10^6^	Motor	14
Ikegame; 2011 [49]	9 / 10	Male; C57BL/6 J; Mice; NR	90	MCAO	0	IV	Allograft	2 × 10^5^	Neuorologic	1
Jiang; 2014 [50]	20 / 20	Male; Sprague-Dawley; Rat; 300–350 g	90	MCAO	0	IA	Autograft	2 × 10^6^	Neuorologic	28
Kim; 2006	9 / 15	Male; Sprague-Dawley; Rat; 250–300 g	Permanent	MCAO	336	IV / IC	Autograft	1 × 10^6^	Motor	70
Leu; 2010 [52]	15 / 15	Male; Sprague-Dawley; Rat; 300–350 g	180	MCAO	24	IV	Allograft	2 × 10^6^	Neuorologic	21
Li; 2016 [53]	6 / 6	Male; Sprague-Dawley; Rat; NR	Permanent	MCAO	24	IV	Autograft	5 × 10^5^	Neuorologic	28
Liu; 2014 [54]	24 / 24	Male; Sprague-Dawley; Rat; 200–250 g	60	MCAO	24	IC	Xenograft	5 × 10^5^	Neuorologic	28
Oh; 2015 [55]	31 / 39	Male; Sprague-Dawley; Rat; 280 to 300 g	90	MCAO	24	IA	Xenograft	5 × 10^5^	Motor/Neuorologic	56
Seo; 2013 [56]	17 / 19	Male/female; CD-1; Mice; NR	90	Carotid clamp	1008	IC	Xenograft	1 × 10^5^	Motor	56
Yang; 2011 [57]	12 / 12	Male; Sprague-Dawley; Rat; NR	60	MCAO	0	IV	Allograft	1 × 10^7^	Motor	28
Yin; 2015 [58]	10 / 10	Male; Sprague-Dawley; Rat; 250–300	90	MCAO	0	IC	Xenograft	1 × 10^4^	Neuorologic	28
Zhao; 2017 [59]	8 / 8	Male; Sprague-Dawley; Rat; 250–320	120	MCAO	0	IV	Allograft	1 × 10^6^	Neuorologic/Motor	28
Zhou; 2015 [60]	7 / 7	Male; C57/BL6; Mice; NR	90	MCAO	168	IC	Xenograft	5 × 10^6^	Motor	49

*IA* Intra-artery, *IC* Intracranial/Intraventricular, *IV* Intravenous, *MCAO* Middle cerebral artery occlusion

### Risk of bias assessment and publication bias

**Fig. 2:**
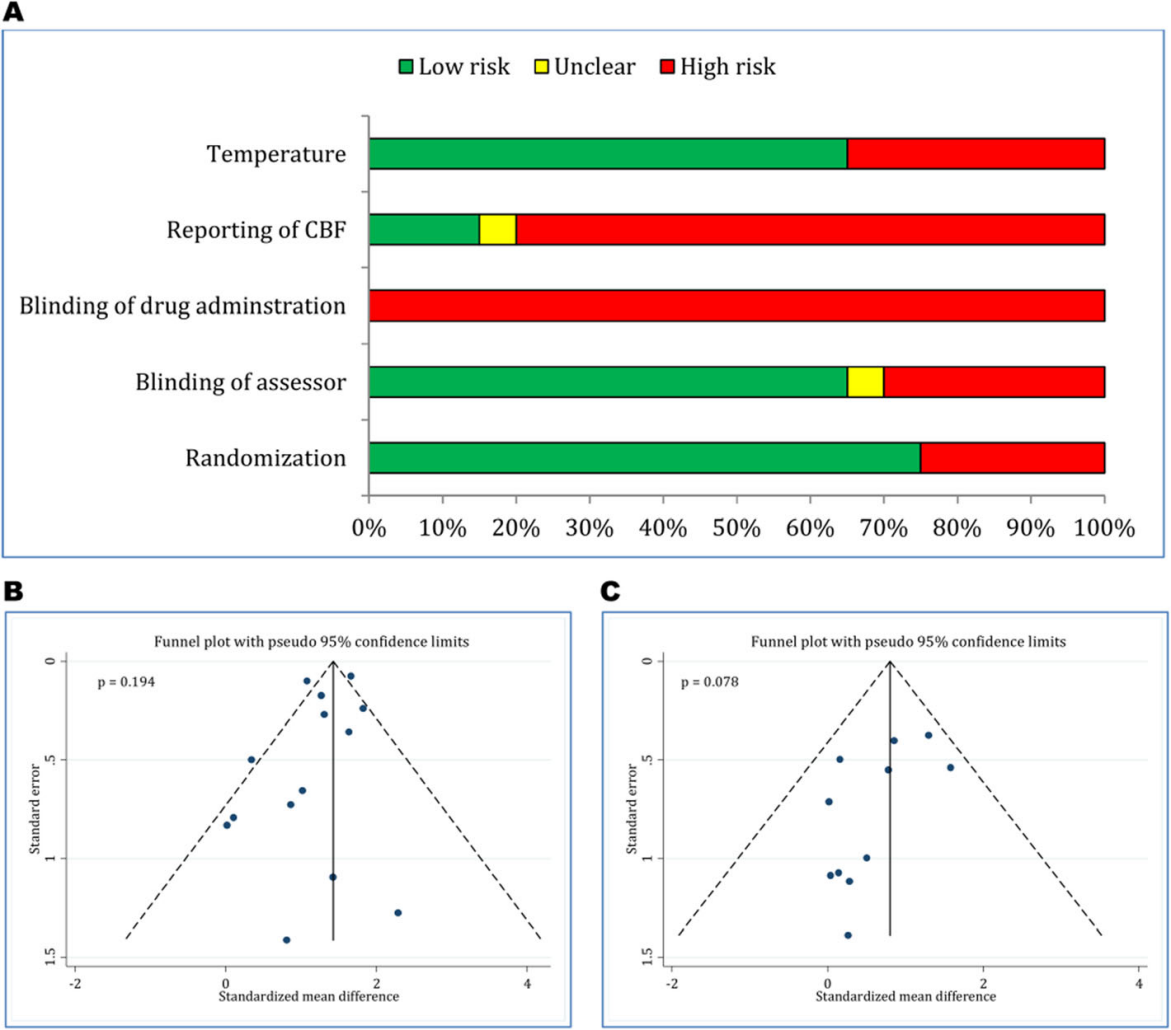
Methodological assessment of included studies (**a**) and risk of bias in evaluation of motor function recovery (**b**) and neurological status (**c**)

Quality control of the articles is presented in Fig. 2a. The quality assessment showed that randomization method was low risk of bias in 75% of the articles. Blinding status of the outcome assessor was low risk of bias in 65% of the articles and 30% of the articles were high risk of bias. Blinding of drug administration in all papers and reporting of cerebral blood flow in 80% of articles was high risk of bias. Reporting of temperature control during the surgical procedure was low risk of bias in 65% of the studies (Table 3). In both sections of functional assessment (*p* = 0.194) and neurological status (*p* = 0.078), no publication bias was observed (Fig. 2b and c).

**Table 3:** Methodological quality assessment of included studies

Author; year	Randomization	Blinding status		Reporting of CBF	Temperature
		Outcome assessed	Cell administration		
**Chen; 2016** [41]	No	Yes	No	No	No
**Chi; 2016** [42]	No	No	No	Yes	No
**Chung; 2015** [43]	No	Unclear	No	Yes	Yes
**Chung; 2017** [44]	Yes	Yes	No	Yes	Yes
**Du; 2014** [45]	Yes	Yes	No	No	Yes
**Ghazavi; 2017** [46]	Yes	Yes	No	Unclear	Yes
**Gutiérrez-Fernández; 2013** [47]	Yes	Yes	No	No	Yes
**Gutiérrez-Fernández; 2015** [48]	Yes	Yes	No	No	Yes
**Ikegame; 2011** [49]	No	Yes	No	No	Yes
**Jiang; 2014** [50]	Yes	Yes	No	No	Yes
**Kim; 2006**	Yes	No	No	No	No
**Leu; 2010** [52]	No	Yes	No	No	No
**Li; 2016** [53]	Yes	No	No	No	Yes
**Liu; 2014** [54]	Yes	Yes	No	No	Yes
**Oh; 2015** [55]	Yes	Yes	No	No	No
**Seo; 2013** [56]	Yes	No	No	No	No
**Yang; 2011** [57]	Yes	No	No	No	Yes
**Yin; 2015** [58]	Yes	No	No	No	Yes
**Zhao; 2017** [59]	Yes	Yes	No	No	Yes
**Zhou; 2015** [60]	Yes	Yes	No	No	No

*CBF* Cerebral blood flow

### Meta-analysis

#### Motor function assessment

**Fig. 3:**
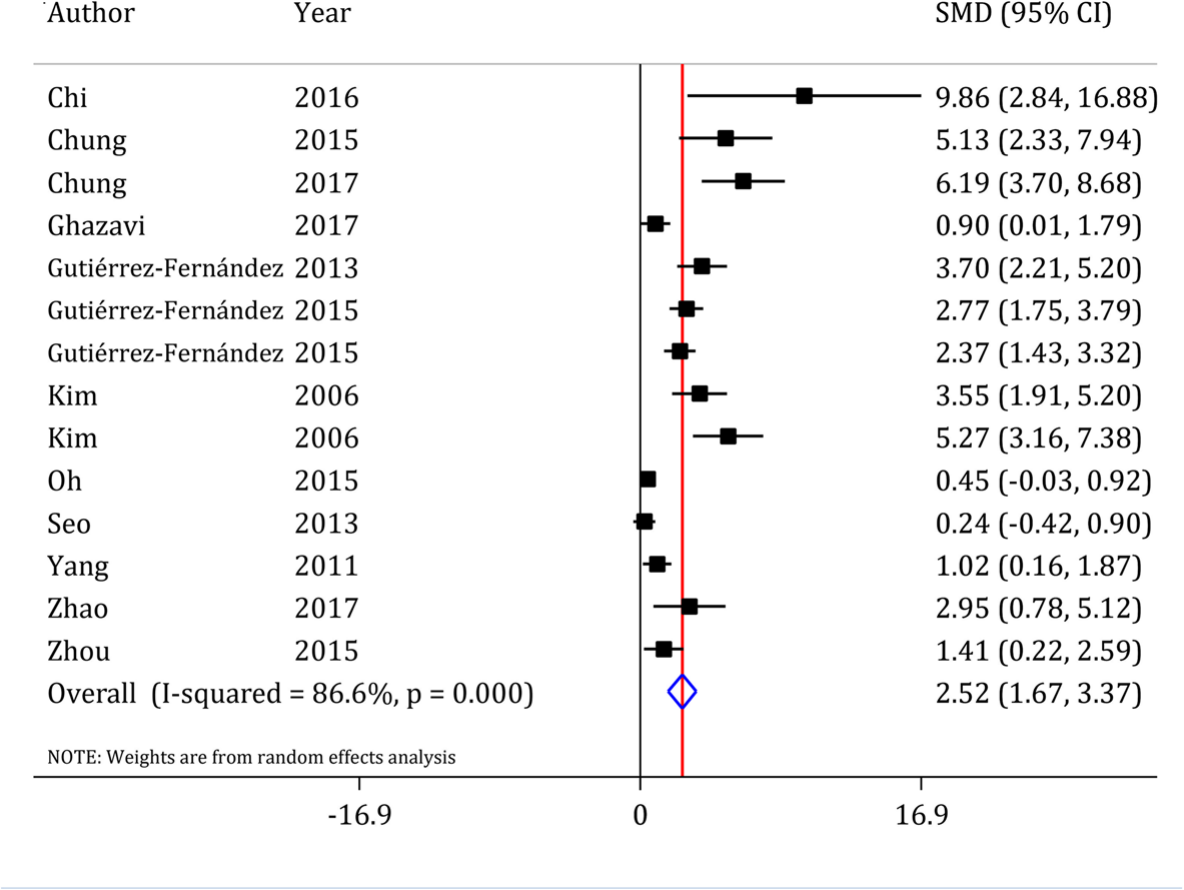
Forest plot of motor function recovery after ADSCs in ischemic stroke. SMD: standardized mean difference; CI: Confidence interval. Gutiérrez-Fernandez et al. 2015, compared xenogeneic and allogeneic adipose mesenchymal stem cells in the treatment of acute stroke. Therefore, two separate experiment were included in the meta-analysis

Eleven studies consisted of 14 separate experiments investigated the effects of ADSCs transplantation on motor function improvement [42–44, 46–48, 51, 55–57, 60]. Pooled analysis showed that administration of ADSCs improves motor function in animals with ischemic stroke (SMD = 2.52, 95% CI: 1.67 to 3.37, *p* < 0.0001; I^2^ = 86.6%, *p <* 0.0001) (Fig. 3). Subgroup analysis showed that ADSCs transplantation in rats improves motor function (*p* < 0.0001), but no significant effect was obsereved in mice (*p* = 0.157). It was also found that ADSCs transplantation (*p* < 0.0001) improves motor function in MCAO model, but does not affect motor function in carotid clamp model (*p* = 0.096). In addition, transcranial (*p* = 0.022) and intravenous (*p* < 0.0001) transplantation improved motor function outcome following stroke in the animals, but intra-arterial administration (*p* = 0.067) had no significant effect on motor function improvement. Administration of ADSCs only affected the motor function with a dose greater than 1 × 10^6^ cells per animal (*p* ≤ 0.001) (Table 4).

**Table 4:** Subgroup analysis of ADSCs transplantation on motor function recovery after stroke

Variable	Effect size		
	SMD (95% CI)	*p* value	Heterogeneity (*p* value)
**Species**			
Mice	1.37 (−0.53 to 3.27)	0.157	79.2% (0.008)
Rat	2.81 (1.82 to 3.80)	< 0.0001	87.4% (< 0.0001)
**Duration of occlusion**			
≤ 60 min	2.85 (0.92 to 4.77)	< 0.0001	86.9% (< 0.0001)
> 60 min	2.48 (1.45 to 3.51)	0.004	87.9% (< 0.0001)
**Stroke model**			
MCAO	2.35 (1.50 to 3.23)	< 0.0001	84.5% (< 0.0001)
Carotid clamp	3.72 (−0.66 to 8.09)	0.096	93.3% (< 0.0001)
**Type of administration**			
Intracranial	2.78 (0.40 to 5.16)	0.022	89.0% (< 0.0001)
Intravenous	2.84 (1.86 to 3.80)	< 0.0001	78.9% (< 0.0001)
Intra-artery	0.45 (−0.03 to 0.92)	0.067	0.00% (> 0.999)
**Type of graft**			
Autograft	4.28 (2.62 to 5.94)	< 0.0001	36.5% (0.209)
Allograft	2.01 (1.00 to 3.02)	< 0.0001	74.5% (0.003)
Xenograft	2.44 (1.13 to 3.74)	< 0.0001	88.7% (< 0.0001)
**Number of transplanted cell**			
1 × 10^5^ to 5 × 10^5^	0.38 (−0.01 to 0.76)	0.057	0.0% (0.620)
1 × 10^6^	4.64 (3.75 to 5.90)	< 0.0001	36.9% (0.160)
2 × 10^6^	2.35 (1.24 to 3.46)	< 0.0001	77.5% (0.004)
5 × 10^6^ to 1 × 10^7^	1.15 (0.46 to 1.84)	0.001	0.0% (0.603)
**Interval time from stroke to treatment**			
0 to 3 h	2.93 (1.86 to 3.99)	< 0.0001	79.8% (< 0.0001)
24 h	NA	NA	NA
More than 7 days	2.44 (0.43 to 4.45)	0.017	90.1% (< 0.0001)
**Follow up duration**			
< 28 days	3.37 (2.0 to 4.68)	< 0.0001	80.8% (< 0.0001)
≥ 28 days	1.76 (0.80 to 2.71)	< 0.0001	84.1% (< 0.0001)

*CI* Confidence interval, *MCAO* Middle cerebral artery occlusion, *NA* Not applicable due to limited number of studies in the subgroup, *SMD* Standardized mean difference

A multivariate meta-regression analysis was performed to assess any possible collinearities in the significant variables in subgroup analyses. The results showed that only stroke model (*p* = 0.017) and dose of transplanted cells (*p* = 0.007) affected the efficacy of ADSCs on motor function improvement following ischemic stroke (Table 5).

**Table 5:** Multivariate meta-regression on efficacy of ADSCs transplantation on motor function recovery after stroke

Variable	Coefficient	95% confidence interval	*p* value
**Species**	−2.71	−6.44 to 1.02	0.135
**Number of transplanted cell**	2.90	1.04 to 4.77	0.007
**Model of stroke**	3.64	0.82 to 6.46	0.017
**Type of administration**	−1.36	−4.61 to 2.95	0.115

#### Neurological status assessment

**Fig. 4:**
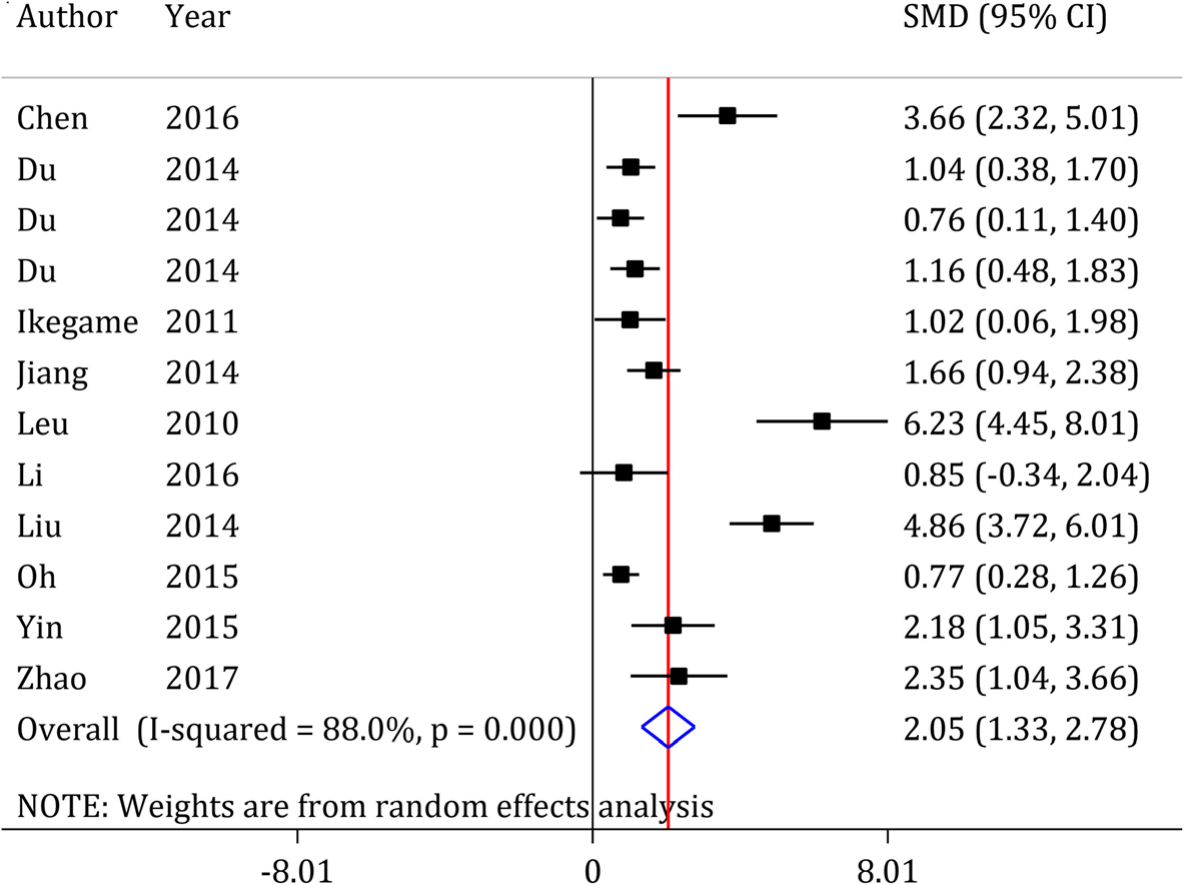
Forest plot of neurological recovery after ADSCs in ischemic stroke. SMD: standardized mean difference; CI: Confidence interval

Nine studies with 12 separate experiments were conducted on the effects of ADSCs transplantation on neurological status of animals with ischemic stroke [41, 45, 49, 50, 52–54, 58, 59]. Pooled analysis showed that administration of ADSCs improves neurological status in animals following ischemic stroke (SMD = 2.05, 95% CI: 1.33 to 2.78, *p* < 0.0001; I^2^ = 88.0%, *p* < 0.0001) (Fig. 4). Furthermore, subgroup analysis showed that none of the variables specie type, duration of occlusion, stroke model, route of administration, type of graft, number of transplanted cells, duration of treatment and follow-up time affect the efficacy of ADSCs transplantation on the improvement of neurological status (Table 6). Since there were no differences among the subgroups, performing a multivariate meta-regression was not applicable for neurological status assessment.

**Table 6:** Subgroup analysis of ADSCs transplantation on neurological improvement after stroke

Variable	Effect size		
	SMD (95% CI)	*p* value	Heterogeneity (*p* value)
**Species**			
Mice	NA	NA	NA
Rat	2.16 (1.38 to 2.94)	< 0.0001	89.0% (< 0.0001)
**Duration of occlusion**			
≤ 60 min	4.32 (3.15 to 5.49)	< 0.0001	43.6% (0.183)
> 60 min	1.56 (0.98 to 2.13)	< 0.0001	79.3% (< 0.0001)
**Stroke model**			
MCAO	2.51 (1.46 to 3.56)	< 0.0001	90.2% (< 0.0001)
Carotid clamp	0.98 (0.60 to 1.36)	< 0.0001	0.0% (0.683)
**Type of administration**			
Intracranial	2.66 (0.42 to 4.90)	0.020	93.8% (< 0.0001)
Intravenous	2.35 (0.98 to 3.71)	< 0.001	89.1% (< 0.0001)
Intra-artery	1.14 (0.63 to 1.65)	< 0.0001	50.8% (0.131)
**Type of graft**			
Autograft	1.40 (0.66 to 2.14)	< 0.0001	22.5% (0.256)
Allograft	1.82 (0.86 to 2.77)	< 0.0001	86.0% (< 0.0001)
Xenograft	2.83 (0.81 to 4.84)	0.006	94.2% (< 0.0001)
**Number of transplanted cells**			
1 × 10^4^ to 5 × 10^5^	1.19 (0.92 to 1.45)	< 0.0001	85.4% (< 0.0001)
1 × 10^6^ to 1.2 × 10^6^	2.99 (2.05 to 3.92)	< 0.0001	47.1% (0.169)
2 × 10^6^	2.31 (1.64 to 2.98)	< 0.0001	95.4% (< 0.0001)
**Interval time from stroke to treatment**			
0 to 3 h	2.07 (1.29 to 2.85)	< 0.0001	63.3% (0.028)
24 h	2.05 (0.99 to 3.10)	< 0.0001	92.0% (< 0.0001)
More than 7 days	No data	No data	No data
**Follow up duration**			
< 28 days	1.74 (0.68 to 2.79)	0.001	87.8% (< 0.0001)
≥ 28 days	2.28 (1.20 to 3.36)	< 0.0001	89.1% (< 0.0001)

*CI* Confidence interval, *MCAO* Middle cerebral artery occlusion, *NA* Not applicable due to limited number of studies in the subgroup, *SMD* Standardized mean difference

## Discussion

The current meta-analysis reviewed preclinical studies that examined the efficacy of ADSCs transplantation as a treatment in ischemic stroke. The findings showed that ADSCs transplantation improves motor function and neurological status following stroke. The degree of recovery in the motor function depends on the stroke model and number of transplanted cells. Since an SMD lower than of 0.2 represents a poor effect for the effect size, 0.5 represents moderate effectiveness and ≥ 0.8 represents a strong effect [61, 62], our meta-analysis results indicate a strong efficacy (SMD = 2.52) of ADSCs on improvement of motor function and neurological status following ischemic stroke.

Comparing our meta-analysis results with other similar meta-analyses (performed evaluating other sources of MSCs) showed that ADSCs had more efficacy in improving motor functional recovery and neurological status than other MSCs. In 2012, Lees and colleagues conducted a systematic review and meta-analysis with the aim of evaluating the effects of MSCs administration in stroke models including and reviewing 117 studies. Of these studies, only two studies used ADSCs, and the rest used other types of MSCs (mostly Bone Marrow Stromal Cells). They showed that the use of MSCs in stroke models can improve structural abnormalities (24·8%) and functional status (40.6%) [63]. In our meta-analysis, all of the included studies used ADSCs, and in addition to functional outcome, neurological status was also assessed.

Vu et al. conducted a meta-analysis in 2014, evaluating the efficacy of different types of MSCs as treatment following ischemic stroke. Of the 46 includes studies in the meta-analysis, 84.8% (39 studies) of studies used bone marrow stromal cells (BMSCs), while 15.2% (7 studies) referred to other types of mesenchymal stem cells (one study used ADSCs, one study used placenta-derived mesenchymal stromal cells, one study was conducted on mesenchymal stem cells derived from peripheral blood and 4 studies used human umbilical mesenchymal stem cells). Their meta-analysis showed that the SMD of MSCs in improving motor function varied from 0.93 to 1.78 [64]. In our study, the efficacy of ADSCs on motor function was 2.52. Pertaining neurological status, our results (SMD = 2.05) showed greater efficacy for ADSCs than Vu et al.’s results (SMD = 1.78) for MSCs. In 2017, Sarmah et al. performed a meta-analysis similar to that of Lees and colleagues. In their analysis, a total of 64 studies were included, of which only 3 studies used ADSCs. The rest of the studies used other types of MSCs (mostly BMSCs) [65]. The results of their study showed that improvement in neurological status (SMD = 0.95 vs. SMD = 2.05) and functional outcome (SMD = 1.07 and 1.04 vs. SMD = 2.5) was less than the results obtained in our study. Also, in 2019, Ouyang et al. performed a meta-analysis in which examined the effects of MSCs transplantation on neurological status and structural outcome following stroke in pre-clinical and clinical studies. In the meta-analysis of pre-clinical studies, 18 studies and in the meta-analysis of clinical studies, 11 studies were included. Of all these studies, the cellular source was ADSCs only in two studies, and the rest of the included studies used different types of MSCs. Their meta-analysis in the pre-clinical section did not assess functional outcome and focused only on neurological status improvement and structural outcomes. The results of their study showed a significant improvement in neurological status and structural outcomes in the cell therapy group [66].

Since most of the included studies in Vu et al., Lees et al., Sarmah et al. and Ouyang et al. meta-analyses were performed on the other types of MSCs (most BMSCs), the higher efficacy, which was observed in our study, can be related to the better biological activity of ADSCs compared to BMSCs in improvement of functional recovery after Stroke. Compared to BMSCs, ADSCs appear to have higher proliferation capacity [67] and migration [68] and colony formation capability [69, 70]. In another meta-analysis, Chen et al. showed that transplantation of neural stem cells (NSCs) could improve locomotor function of animals after stroke and can reduce the infarct size [71]. Although neural stem cells are the closest cell type to the injured nervous tissue, and thus may be a better source for treatment of central nervous system diseases, they are difficult to extract, which is a serious limitation for their use in the treatment process of the patients. An alternative source is induced pluripotent stem cell (IPSCs) derived NSCs (IPSCs-derived NSCs). IPSCs-derived NSCs do not have ethical considerations and their immune rejection risk is the lowest. In addition, recent studies showed that IPSCs-derived NSCs present a stable neural phenotype and can differentiate to neural cells. However, there are several concerns over the use of IPSCs including tumorigenesis, high rate of apoptosis, sensitivity to ionization radiation, insertional mutagenesis and etc. [72]. Therefore, well-design studies are needed to assess the safety and efficacy of IPSCs as a treatment option for ischemic stroke [37].

In our study, the efficacy of ADSCs was not affected by the route of administration. In a systematic review and meta-analysis in 2017, Nagpal et al. concluded that the transplantation of stem cells can improve functional recovery. However, the researchers were not able to determine the best route and the optimal dose of stem cells administration in the treatment of stroke [73]. As a result, the intravenous administration seems to be more appropriate for treating stroke patients, since the intracranial injection is an invasive method [64].

Vu et al. in 2014 showed that the efficacy of MSCs in the treatment of stroke is dose-dependent [64]. Therefore, determining the optimal dosage for administration of MSCs in order to treat stroke is a challenging factor. The results of the current meta-analysis showed that the efficacy of ADSCs on motor function improvement is higher when it is transplanted in a dose of equal or higher than 1 × 10^6^ cells. In addition, the neurological improvement was observed in all transplanted doses of ADSCs. Therefore, it seems that the optimum dose of ADSCs, which can improve the motor function and neurological status, is 1 × 10^6^ per animal.

In the motor function assessment, we observed an extreme efficacy for ADSCs administration after ischemic stroke. Chi et al. [42] showed that the transplantation of ADSCs has a great improving effect on motor function following stroke. The extreme efficacy reported in Chi et al. study may be due to the use of permanent stroke model. The permanent stroke causes more severe injury and higher motor/neurologic deficiency compared to transient ischemic stroke model. Therefore, it is predictable that the difference between the treated and untreated groups in this study is greater than the difference in other studies.

The follow up duration varied between 1 and 70 days among eligible studies. Obviously, some degrees of functional/neurological improvement following stroke may be observed after this time. This improvement is time dependent. So, longer follow up duration may be associated with higher improvement rates following stroke. However, the meta-regression showed that duration of follow up does not affect the efficacy of ADSCs on motor and neurological improvement. This finding may be due to the fact that most of the studies transplanted the ADSCs during the acute and subacute phases following stroke. In the acute phase following ischemic stroke, sudden loss of blood circulation initiate a cascade of pathophysiologic response, which can last from days to several weeks [74]. Therefore, transplantation of ADSCs in this time could prevent the pathologic changes after stroke through its paracrine secretory characteristics. For example, previous studies showed that ADSCs transplantation after ischemic stroke decrease neuronal loss, prevents blood-brain barrier disruption and reduces neuronal oxidative injury [43, 44]. In other words, ADSCs could reduce the inflammatory responses after ischemic stroke and facilitate neuronal regeneration and revascularization in the peri-infarct region [75]. Therefore, transplantation of ADSCs at the early phase of ischemic stroke could reduce apoptosis and the infarct volume and thus, significantly improve motor/neurologic status during the first hours after injury [46].

Univariate subgroup analysis showed that ADSCs transplantation in mice does not improve motor function. This difference in response to the treatment between rats and mice may be due to co-linearity of some other experimental feature with animal spices. Therefore, we fitted a multivariate meta-regression, and the results confirmed the hypothesis. In the multivariate meta-regression analysis, animal species type has no effects on the efficacy of ADSCs on motor function improvement following ischemic stroke.

### Limitations and suggestions

Overall, the quality control of the studies included in the current meta-analysis shows that all of these studies did not estimate the sample size and the optimal dosage, which are important factors in clinical studies [62]. In addition, the main goal in animal studies is to evaluate the efficacy of the treatment but in the clinical trials, before the efficacy, the safety of a treatment is considered, which is not considered in animal studies. Only one study assessed the safety of ADSCs in treatment of stroke [48]. Therefore, a meta-analysis could not be performed about the safety of the treatment. So, one of the most serious limitations in the present meta-analysis, like most studies conducted on animal models, is the lack of safety assessments.

On the other hand, a considerable heterogeneity was observed when evaluating the efficacy of ADSCs in improving motor function and neurological status. Subgroup analysis led to the determination of the source of heterogeneity in the motor function, but its origin was not determined in the neurological status. Therefore, the level of evidence presented in the neurological status is one step lower and considered as moderate.

Finally, there are no reports regarding the dose-response effect of ADSCs on the improvement of ischemic stroke in the literature. Although we performed a subgroup analysis in different doses of ADSCs on sensory-motor improvement following stroke, the lack of evidence on dose-response effect, raises the concern over the efficacy of ADSCs treatment which may not be biologically plausible. Further pre-clinical studies are recommended to assess the dos-response effect of ADSCs on sensory-motor function following an ischemic stroke.

## Conclusion

Moderate to high levels of evidence indicate a strong efficacy of ADSCs transplantation on motor function and neurological improvement following ischemic stroke in animal models. This treatment is more effective in focal ischemic models than in global stroke. It was also found that the optimum number of transplanted ADSCs is 1 × 10^6^ per animals. However, there are no reports regarding the dose-response effect of ADSCs on stroke in the literature. Thus, further pre-clinical studies are recommended.

## Supplementary information

**Additional file 1: Figure S1.** Meta-regression for effect of follow up duration on efficacy of adipose tissue derived stem cells (ADSCs) on motor (A) and neurologic (B) improvement after ischemic stroke. Analyses showed duration of follow up does not affect the efficacy of ADSCs on motor and neurologic improvement. Coef.: Meta-regression coefficient; CI: Confidence interval.

## Data Availability

All data generated or analyzed during this study are included in this published article.

## Abbreviations

95% CI: 95% confidence interval
ADSCs: Adipose derived stem cells
BMSCs: Bone marrow stromal cells
IPSCs: Induced pluripotent stem cell
MSCs: Mesenchymal stem cells
MCAO: Middle cerebral artery occlusion
NSCs: Neural stem cells
SMD: Standardized mean difference
