# Supplementary material for: Efficacy of adipose derived stem cells on functional and neurological improvement following ischemic stroke: a systematic review and meta-analysis
Source: BMC Neurol. 2020 Aug 10;20:294. doi: 10.1186/s12883-020-01865-3 (PMC7418438; doi:10.1186/s12883-020-01865-3)
Supplement: Supplementary file 1 — Additional file 1: Figure S1. Meta-regression for effect of follow up duration on efficacy of adipose tissue derived stem cells (ADSCs) on motor (A) and neurologic (B) improvement after ischemic stroke. Analyses showed duration of follow up does not affect the efficacy of ADSCs on motor and neurologic improvement. Coef.: Meta-regression coefficient; CI: Confidence interval. [file 12883_2020_1865_MOESM1_ESM.docx]

| **A** | **B** |
| --- | --- |
| **** | **** |

Figure S1: Meta-regression for effect of follow up duration on efficacy of adipose tissue derived stem cells (ADSCs) on motor (A) and neurologic (B) improvement after ischemic stroke. Analyses showed duration of follow up does not affect the efficacy of ADSCs on motor and neurologic improvement. Coef.: Meta-regression coefficient; CI: Confidence interval.
